# A study on the position and etiology of infection in cirrhotic patients: A potential precipitating factor contributing to hepatic encephalopathy

**DOI:** 10.3892/etm.2013.1137

**Published:** 2013-06-03

**Authors:** QIU-MING WANG, QING JI, ZHI-JUN DUAN, MING ZHANG, QING-YONG CHANG

**Affiliations:** 1 Department of Gastroenterology, First Affiliated Hospital of Dalian Medical University, Dalian, Liaoning 116011;; 2 Department of Gastroenterology, Shiyan Taihe Hospital, Shiyan, Hubei 44200;; 3 The Second Affiliated Hospital of Xi’an Medical College, Xi’an, Shaanxi 710038;; 4 Department of Neurosurgery, Zhongshan Affiliated Hospital of Dalian University, Dalian, Liaoning 116001, P.R. China

**Keywords:** hepatic encephalopathy, precipitating factors, infection

## Abstract

Hepatic encephalopathy (HE) is a severe and high-mortality complication in cirrhotic patients. In this study, we analyzed infection, one of the common precipitating factors of HE in patients with cirrhosis, in order to identify common infection sites and the etiology. In addition, we aimed to identify information useful in the early prevention and effective treatment of HE. Ninety-two patients presenting with hepatitis B virus-related cirrhosis with HE (HBC-HE) and 45 patients presenting with alcoholic cirrhosis with HE (ALD-HE) were enrolled in this study. We collected and analyzed data concerning the precipitating factors of HE using blood tests, biochemical detection and bacterial culture to identify which precipitating factor was the most common. Fifty-three patients with HE (37 with HBC-HE and 16 with HBC-HE) had infection as the precipitating factor. These infections included respiratory tract infection (56.6%), intestinal tract infection (20.7%), peritoneal infection (17.0%) and urinary tract infection (5.7%). The white blood cell (WBC) counts increased in 17 cases (32.1%) and neutrophil (NEUT) numbers increased in 39 cases (73.6%), while WBC counts were lower in the patients with respiratory tract infection compared with those in the patients with infections at other sites (P<0.05). The levels of plasma ammonia were significantly higher in patients with intestinal tract infection than in those with other sites of infection (P<0.05). The proportions of patients with hyperammonemia, increased NEUT numbers, hyponatremia and low albumin were higher in the infection group compared with those in the non-infection group (P<0.05). Pneumococci and *E. coli* were common bacteria that induced infection in the respiratory tract and at other infection sites, respectively. Respiratory tract infection was identified to be the most common precipitating factor for HE.

## Introduction

Hepatic encephalopathy (HE) is a clinical neuro-psychiatric syndrome based on metabolic disturbances and caused by severe liver diseases and/or portal-systemic shunts. The World Congress of Gastroenterology (WCOG) in Vienna classified HE into types A, B and C depending on the nature of hepatic disturbances or dysfunctions (1). Type A describes HE associated with acute liver failure and type B includes HE associated with portal-systemic bypass. Type C, the most common, is HE associated with cirrhosis and portal hypertension and/or portal-systemic shunts, and may also be subdivided into minimal hepatic encephalopathy (MHE) and symptomatic hepatic encephalopathy (SME) according to the clinical manifestations.

Alcoholic liver disease is common in certain countries, but viral hepatitis-associated cirrhosis is more prevalent in China. Notably, the incidence of drug-induced liver disease is increasing (2). Many patients may develop fibrosis, or even cirrhosis, and may subsequently develop certain complications of cirrhosis, including HE.

There are a number of potential precipitating factors contributing to HE in patients with end-stage cirrhosis. The most common precipitating factor is gastrointestinal bleeding, which leads to the increased production of proteins and, consequently, the increased production of nitrogenous products, particularly ammonia, in the gut. Other precipitating factors include constipation, excessive dietary protein, shock, hyponatremia, hypokalemia, alkalosis and certain sedatives (3). In the majority of cases, HE is caused by a single precipitating factor. Treatment or removal of the precipitating factor may allow the patient to recover from HE within 12–24 h. Shawcross *et al* observed that infection is a frequent precipitating factor of HE in cirrhosis and also revealed an association between infection and systemic inflammation, but not ammonia, in cirrhotic patients that develop severe HE (4).

Patients with serious liver disease often acquire serious infections and endotoxic blood diseases, including spontaneous bacterial peritonitis (SBP), sepsis, pneumonia and urinary tract infections, due to a weakened mononuclear macrophage system. These infections and endotoxic diseases aggravate liver damage and increase ammonia production. Bacterial infection is a main cause of mortality in cirrhotic patients with HE (5). At present, the removal of the precipitating agent is regarded as the primary approach for preventing HE. In the current study, 137 patients had clear precipitating factors of HE and the encephalopathy subsided following the resolution of the precipitating problem. A number of studies have focused on the infection precipitating HE, however, the sites of infection remain controversial (4,6,7). Our previous study demonstrated that infection was a common precipitating factor for the development of HE in patients with hepatitis B virus-related cirrhosis (HBC) (8). In the present study, we aimed to clarify the differences in the main infection sites between HE patients with cirrhosis induced by hepatitis B virus and those with cirrhosis induced by alcohol.

In this study, we analyzed infection as a precipitating factor of HE, in order to identify the most common infection site and the etiology. The findings may aid in the early diagnosis and prevention of HE and the identification of effective treatments.

## Materials and methods

### Patients

A total of 92 inpatients with HE caused by HBC, who were treated at the Department of Gastroenterology (First Affiliated Hospital of Dalian Medical University, Dalian, China) were enrolled in the HBC-HE group according to the exclusion criteria. From March 2003 to October 2012, 45 inpatients with HE caused by alcoholic liver disease (ALD) were selected as a control group (ALD-HE). The patients in the two groups received the same treatment, regained recognizable signs of awareness following the administration of therapies including oxygen inhalation, hepatoprotectants, diuretics and antiencephalopathic agents, and complications were prevented.

The diagnosis of HBC and alcoholic cirrhosis were based on patient history, physical examination, liver imaging and laboratory findings. The diagnosis of alcoholic cirrhosis was also carried out according to the Alcoholic Liver Disease Diagnosis and Treatment Guidelines (9). The diagnosis of HE was made according to the recommendations of the working party of the 11th World Congress of Gastroenterology and the diagnosis of HE grades was based on the West Haven criteria (1). The investigation conformed to the principles outlined in the Declaration of Helsinki and was approved by the ethics committee of The First Affiliated Hospital of Dalian Medical University. All participants in the study provided informed written consent.

### Exclusion criteria

The patients were excluded if they had the following: i) severe cerebrovascular, heart, lung or renal disease, intracranial tumor or infection; ii) craniocerebral disease, epilepsy, psychosis, coma or mental disorder due to other causes; iii) metabolic or toxic encephalopathy (e.g., uremia, hypoglycemia or diabetes); iv) mortality following treatment; v) abdominal surgery history (e.g., upper digestive tract sclerosis surgery, splenectomy, portal-systemic shunt or portal-systemic disconnection) or had undergone treatments using an artificial liver support system or for hepatic cancer.

### Data collection

We collected and classified the clinical data including gender, age and precipitating factor of HE. The laboratory test report further confirmed the sites of infection. White blood cell (WBC) counts, neutrophil (NEUT) numbers, plasma ammonia levels and bacterial culture results were also recorded in this study.

### Statistics

SPSS 11.5 software (SPSS, Inc., Chicago, IL, USA) was used for statistical analysis. Measurement data and qualitative data were expressed as mean ± standard deviation (SD) and frequency, respectively. Differences between the two groups were evaluated by an independent sample Student’s t-test. Percentages were used to analyze constituent ratio and a χ^2^ test was used for qualitative data. P<0.05 was considered to indicate a statistically significant result.

## Results

### Incidence of HE precipitating factors in patients with cirrhosis

**Figure 1.**
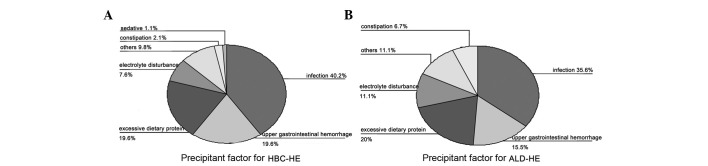
Precipitating factors of cirrhosis with HE. (A) Infection was the most common precipitating factor in the HBC-HE group (40.2%). (B) Infection was also the primary precipitating factor in the ALD-HE group. HE, hepatic encephalopathy; HBC-HE, hepatitis B virus-related cirrhosis with hepatic encephalopathy; ALD-HE, alcoholic cirrhosis with hepatic encephalopathy.

Analysis of the proportions of various precipitating factors in the HBC-HE group showed that infection was the most common precipitating factor (40.2%), followed by upper gastrointestinal hemorrhage (19.6%), excessive dietary protein (19.6%), electrolyte disturbance (7.6%), constipation (2.1%), certain sedatives (1.1%) and others (9.8%; Fig. 1A).

In the ALD-HE group, infection was observed to be the primary precipitating factor (35.6%), followed by upper gastrointestinal hemorrhage (15.5%), excessive dietary protein (20%), electrolyte disturbance (11.1%), constipation (6.7%) and others (11.1%; Fig. 1B).

In the infection group Child-Pugh class A, B and C accounted for 12, 30 and 11 patients, respectively. Ascites and splenomegaly were observed in 44 (83.02%) and 48 patients (90.57%), respectively. These were significantly different to the incidences in the non-infection group (P<0.05; Table I). Infection was the precipitating factor for 37 cases (40.2%), in the HBC-HE group and 16 cases in the ALD-HE group (35.6%; P>0.05). Gender, age and the incidence of gastroesophageal varices were not significantly different between the infection and non-infection groups (Table I).

**Figure 2.**
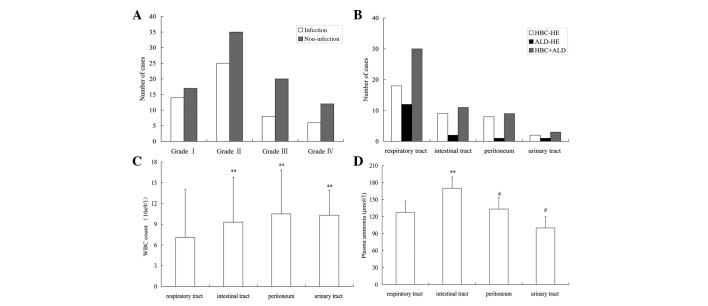
(A) Grades of HE in cirrhotic patients in the infection and non-infection groups. (B) The respiratory tract was the primary infection site in patients with HE (P<0.01) and the intestinal tract was a common infection site compared compared to the other sites (P<0.01; n=53). (C) The WBC count was lower in the patients with respiratory tract infection than in those with infection at other sites (P<0.01; n=53). (D) Comparison of plasma ammonia levels in patients with different infection sites. HE, hepatic encephalopathy; WBC, white blood cell; HBC-HE, HE due to hepatitis B virus-related cirrhosis; ALD-HE, HE due to alcoholic liver disease; ^**^P<0.01 vs. respiratory tract; ^#^P<0.01 vs. intestinal tract.

The grades of HE according to the West Haven criteria were grade I in 14 (26.4%), grade II in 25 (47.2%), grade III in 8 (15.1%) and grade IV in 6 (11.3%) patients in the infection group. In the non-infection group, grade I HE was observed in 17 patients (20.2%), grade II in 35 (41.7%), grade III in 20 (23.8%) and grade IV in 12 (14.3%; Fig. 2A). There were no significant differences in HE grades among patients with different infection sites (P=0.233; Table II).

### Distribution of infection sites in cirrhosis with HE

There were 53 patients with HE who had infection as a precipitating factor. The infections were observed in the respiratory tract in 30 cases (56.6%), intestinal tract in 11 cases (20.7%), peritoneum in 9 cases (17.0%) and urinary tract in 3 cases (5.7%; Fig. 2B). The number of intestinal tract infections was increased compared with peritoneum infections (P<0.01; Fig. 2B). The number of patients who presented with infection at one of the four sites was greater in the HBC-HE group than in the ALD-HE group, however the difference was not statistically significant (P>0.05).

For the HBC-HE and ALD-HE groups, the incidences of respiratory tract infection (n=18, 48.6% and n=12, 75%, respectively), intestinal tract infection (n=9, 24.3% and n=2, 12.5%, respectively), peritoneal infection (n=8, 21.6% and n=1, 6.2% respectively) and urinary tract infection (n=2, 5.4% and n=1, 6.2%, respectively) were not statistically significant between the groups (P>0.05; Fig. 2B).

### WBC count and plasma ammonia in cirrhosis with HE with different infection sites

The comparison of WBC counts in HE patients with different infection sites determined by blood cell analysis showed that the WBC count was significantly lower in patients with respiratory tract infection compared with those in patients with infections in the intestinal tract (P=0.037), peritoneum (P=0.029) and urinary tract (P=0.032). There were no significant differences of WBC count among patients with intestinal tract, peritoneum and urinary tract infections (P>0.05; Fig. 2C).

There were 53 (38.7%) cases with infection as a precipitating factor out of a total of 137 cases as determined by blood cell analysis, including cases with increased WBC counts (n=17, 32.1%), normal WBC counts (n=24, 45.3%) and decreased WBC counts (n=12, 22.6%). The majority of the 53 patients with infection had an increased neutrophil count (NEUT; n=39, 73.6%). Compared with the non-infection group, the infection group had a significantly greater incidence of increased NEUT count cases (χ^2^=59.173, P<0.0001; Table III).

Notably, the incidence of hyperammonemia in the infection group was higher than that in the non-infection group (P= 0.015; Table III). The levels of plasma ammonia were greatly increased when the infection was in the intestinal tract compared with when it was in the peritoneum, respiratory tract or urinary tract (Fig. 2D).

Hyponatremia and low albumin were more common in the infection group (75.47 and 79.25%, respectively) than in the non-infection group (46.43 and 63.1%, respectively; Table III). However, there were no significant differences between the proportions of cases with hyperbilirubinemia and long prothrombin time (PT) between the two groups (P>0.05; Table III).

### Etiology of infection at different sites

Pneumococci and *Pseudomonas aeruginosa* were the most commonly identified pathogenic bacteria (40 and 53.33%, respectively) in respiratory tract infection in liver cirrhotic patients with HE. *E. coli* was the most prevalent pathogenic bacteria identified in the intestinal tract, peritoneum and urinary tract infections in these patients (Table IV).

## Discussion

HE is a common complication of posthepatitic cirrhosis and severe hepatitis, and it is the most common cause of mortality in cirrhotic patients (10). HE may be induced by a variety of factors and the recognition of precipitating factors and their correction or early removal is regarded as the primary approach for improving prognosis and reducing mortality.

In the current study, infection was a common precipitating factor, in addition to upper gastrointestinal hemorrhage, excessive dietary protein, electrolyte disturbance, constipation and sedatives. A previous study also showed that infection and upper gastrointestinal hemorrhage were primary precipitating factors in patients with HE (11). We observed that infection was a main precipitating factor for HE, with an incidence of 40.2% in the HBC-HE group and 35.6% in the ALD-HE group. It has been reported that upper gastrointestinal hemorrhage is the most common precipitating factor in cirrhotic patients (12). However, infection was the most common precipitating factor identified in the present study. This difference may be due to dietary habits, living environment and etiology in different regions. In our routine treatment, a prevention strategy for HE was often performed, so the incidence of HE in patients with upper gastrointestinal hemorrhage was less than that which would otherwise occur. In addition, only HBC-HE and ALD-HE patients were selected for this study, so the results may have limited etiology. In addition, we also observed the proportion of upper gastrointestinal hemorrhage was 19.6% in the HBC-HE group and 15.5% in the ALD-HE group. Portal hypertension may occur earlier in HBC-HE group than in the ALD-HE group and lead to increased upper gastrointestinal hemorrhage. The lower number of ALD-HE cases may be another reason for the difference in results.

**Figure 3.**
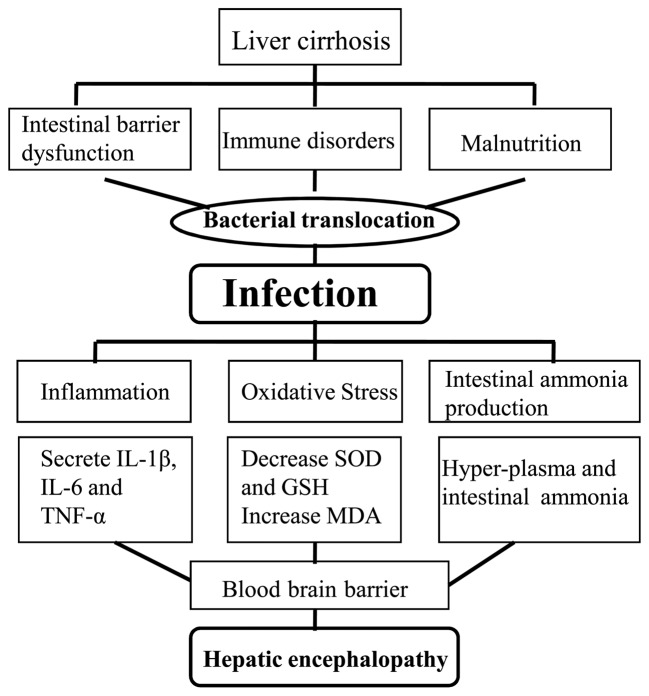
Infection-induced hepatic encephalopathy in liver cirrhosis. Patients with liver cirrhosis develop intestinal barrier dysfunction, immune disorders and malnutrition due to portal hypertension. Bacteria then invade the body via the respiratory, intestinal and urinary tracts and result in various infections. Infection may activate inflammatory reaction and oxidative stress, and cause the secretion of several cytokines, including IL-1β, IL-6 and TNF-α. Reduced cerebral SOD levels and increased production of intestinal ammonia is observed in patients with HE. These inflammatory mediators selectively cross the blood-brain barrier and lead to hepatic encephalopathy (brain injury, edema). IL-1β, interleukin-1β; TNF-α, tumor necrosis factor-α; SOD, superoxide dismutase; GSH, glutathione; MDA, malondialdehyde; HE, hepatic encephalopathy.

Oxidative/nitrosative stress and a low-grade cerebral edema are key events in the pathogenesis of ammonia toxicity and hepatic encephalopathy (13). Patients with advanced liver disease are susceptible to infection due to a dysfunction of host defense mechanisms (14). Infection/systemic inflammatory response has been reported to contribute to the exacerbation of HE in patients with acetaminophen-induced acute liver failure (15). The peripheral immune system normally produces various pro-inflammatory cytokines, including interleukin-1β (IL-1β), IL-6 and tumor necrosis factor-α (TNF-α) during infection. These peripheral cytokines may either directly cross the blood-brain barrier or indirectly signal the brain to interact with circumventricular organs, and activate afferent neurons of the vagus nerve via other informational substances (16). Another study clarified that IL-1 or TNF-α receptor gene deletions delayed the onset of encephalopathy and attenuated brain edema in experimental acute liver failure (17). In addition, mild hypothermia resulted in reduced expression of circulating proinflammatory cytokines, improved neurological function, normalized glutathione levels and attenuated hepatic damage (17). This suggests that infection/systemic inflammatory response is a key factor contributing to HE (Fig. 3).

Non-steroidal anti-inflammatory drugs (NSAIDs), such as ibuprofen, may reduce hypokinesia and microglial activation, thereby restoring normal motor activity and cognitive function in rats with HE (18,19). These results may further support the role of inflammation in the induction of HE.

In addition, patients with liver cirrhosis are abnormally susceptible to infection as a result of immunological deficits. The mechanisms of action include reduced hepatic production of complement (reduced C3 and C5 levels), impaired phagocytosis of Kupffer cells and clearance of inflammatory cytokines, and altered neutrophil chemotaxis (20). In the liver, the function of reticuloendothelial cells is to remove bacteria from the blood. The activation of macrophages in cirrhosis is dysfunctional and the sterilization ability reduced, leading to a dysfunction of the reticuloendothelial system. Shawcross *et al* demonstrated that downregulation of HLA-DR expression on monocytes resulted in immunogical deficits in decompensated liver cirrhosis (21).

Astrocytes and endothelial cells are able to release a variety of inflammatory cytokines, which lead to intracranial hypertension and brain edema when the body is in infection status. NSAIDs have been reported to ameliorate intracranial hypertension and brain edema in patients and rats with HE due to a portacaval shunt (22).

Shawcross *et al* (23) proposed that systemic inflammation exacerbates the neuropsychological alterations induced by hyperammonemia. Previous studies have shown that inflammation and ammonia act synergistically to exacerbate HE symptoms (24–26). Ammonia contributes to neutrophil swelling and phagocytosis dysfunction (27). In the current study, we observed that the majority of cirrhotic patients with HE due to infection had hyperammonemia. In addition, the levels of plasma ammonia were greatly increased in patients with intestinal tract infection compared with those in patients with infections at other sites. We suggest that intestinal tract infection may lead to hyperammonemia in these patients by increasing the production of intestinal ammonia. Neutrophils are an important component of the body’s immune system. High levels of ammonia have been demonstrated to induce neutrophil swelling and impaired neutrophil phagocytosis, resulting in neutrophil dysfunction in rats fed an ammoniagenic diet and in patients with cirrhosis (28). Patients with acute-on-chronic liver failure had a similar degree of depression of cellular immunity and monocyte levels, which contributed to the increased infection morbidity of these patients (29,30). In a previous study, Child-Pugh class C cirrhotic patients that presented with downregulation of HLA-DR expression on monocytes exhibited immune dysfunctions (31). Consequently, infection-enhancing catabolism led to an increasing production of ammonia; therefore, the prevention and control of infection may reduce the incidence of HE in cirrhotic patients.

The incidence of infection in patients with HBC-HE and ALD-HE remains uncertain. With a group of 382 cirrhotic inpatients, Rosa *et al* (32) demonstrated that alcoholic patients with Child-Pugh class A/B disease were more susceptible to infection compared with nonalcoholic patients, but in those patients with Child-Pugh class C disease, no statistical difference was observed in the infection or mortalities between alcoholics and nonalcoholics. Borzio *et al* (33) demonstrated that the infection rate was not statistically different between alcoholic patients and HBC-HE patients with advanced cirrhosis, and was associated more closely with the severity than with the etiology of the hepatic disease. There was no statistical difference in the incidence of infection between the HBC-HE and the ALD-HE groups in this study. This may be due to the relatively smaller number of alcoholic patients (45 cases) enrolled, and therefore a larger number of cases should be collected for further study. The mechanism by which alcoholic patients become more susceptible to infection and endoxemia may be through alcohol-induced damage to the immune system.

A previous study reported that spontaneous bacterial peritonitis (SBP) is currently the most frequent infection in cirrhosis (6). A domestic clinical study revealed that pulmonary infection was the most common, followed by peritoneal infection and urinary tract infection (7). In a total of 53 cases of HE with infection in the current study, we observed that respiratory tract infection (n=30, 56.6%) was the primary precipitating factor, followed by intestinal infection (n=11, 20.7%), peritoneal infection (n=9, 17.0%) and urinary tract infection (n=3, 5.7%). The result was not in accordance with the findings of previous studies which reported that SBP was the primary precipitating factor in cirrhotic patients with HE due to infection. This may be due to differences in etiologies, Child-Pugh classes, treatments, regions and ethnic origins. Bacterial overgrowth, intestinal motility and barrier dysfunction were conducive to bacterial translocation leading to bacteremia. The mononuclear phagocyte system is suppressed and the ability of the liver to remove bacteria declines, leading to reductions in immunoglobulin, complement and albumin and ultimately results in ascites infection (34). The risk for SBP in cirrhotic patients with ascites was associated with Child-Pugh class and patients with Child-Pugh class C disease were susceptible to peritoneal infection (35). A greater number of Child-Pugh class A/B patients and fewer Child-Pugh class C patients were enrolled in our study. In addition, a high proportion of grade II (47.2%) patients with HE had infection as a precipitating factor.

A significantly lower WBC count was observed in the patients with respiratory tract infection than in patients with infection at other sites. Respiratory infection included upper and lower respiratory tract infection, and viral infection was more common in the upper respiratory tract, which is likely to cause the WBC count to decrease. However, in the current study, the intestinal tract, peritoneum and urinary tract presented with more bacterial infections, which led to an increased WBC count. The WBC count increased in 32.1% of the patients with HE due to infection in our study. In addition, NEUT counts were significantly different between the infection and non-infection cases and, as expected, the NEUT count increased in infection cases. Fewer patients with bacterial infection had a high WBC count, due to immune dysfunction and hypersplenism. Therefore, doctors may not make a diagnosis on the basis of WBC count changes and left shift should be observed in cirrhotic patients with complicated infectious diseases.

Shawcross *et al* also observed that grade III/IV encephalopathy correlates with the presence of systemic inflammatory response syndrome (SIRS) and not with ammonia (4). We observed in the present study that the grade of encephalopathy had no significant association with different infection sites.

Ascites, hyponatremia and low albumin were common in the infection group. Cirrhotic patients with ascites are susceptible to bacterial infection which develops into SBP and is predominantly caused by enteric organisms (36). Hyponatremia is common in patients with cirrhosis and correlates with the complications of cirrhosis, including hepatorenal syndrome, encephalopathy and SBP (37). In cirrhotic patients with ascites, ∼50% have a certain degree of hyponatremia (38). Hyponatremia complicates the management of cerebral edema and increases the risk of developing or exacerbating HE (39). Serum sodium and ammonia levels are major factors determining electroencephalographic abnormalities in cirrhosis (40). In a previous study, serum sodium was an independent predictive factor of HE in patients with cirrhosis (41). In addition, there was a close association between hyponatremia and HE, which was in accordance with other studies (39).

Enteric gram-negative cocci have been reported to be the microorganisms most frequently isolated in SBP patients with hospital-acquired infections (42). We observed that *E. coli* was a prevalent bacteria in intestinal tract, peritoneum and urinary tract infections.

In conclusion, respiratory infection was a common precipitating factor for HE in patients with cirrhosis. The WBC count was not always increased in cirrhotic patients with HE induced by infection. An increased WBC count combined with left shift may indicate the occurrence of infection. As a retrospective analysis, the number of cases was inadequate and the study lacked patients with other types of cirrhosis. The control of respiratory tract infection is pivotal in the prevention and treatment of HE in the future. Mortality from HE is likely to decrease due to early diagnosis, the use of appropriate antibiotic therapy and albumin administration.

## Figures and Tables

**Table I.** Comparison of clinical features between the infection and non-infection groups.

Clinical feature	Infection (n=53)	Non-infection (n=84)	P-value
HBC-HE/ALD-HE	37/16	55/29	0.599
Gender (male/female)	36/17	53/31	0.564
Age (years)	60.01±11.99	67.23±10.32	0.187
Child-Pugh class (A/B/C)	12/30/11	39/33/12	0.007
Ascites, n (%)	44 (83.02)	53 (63.10)	0.012
Gastroesophageal varices, n (%)	39 (73.58)	60 (71.43)	0.784
Splenomegaly, n (%)	48 (90.57)	56 (66.67)	0.001

Age data are mean ± standard deviation (SD). HBC-HE, hepatitis B virus-related cirrhosis with hepatic encephalopathy; ALD-HE, alcoholic cirrhosis with hepatic encephalopathy.

**Table II.** Comparison of hepatic encephalopathy (HE) grades in patients with different sites of infection.

Infection site	n	Grade I (n=14) n (%)	Grade II (n=25) n (%)	Grade III (n=8) n (%)	Grade IV (n=6) n (%)	P-value
						0.233
Respiratory tract	30	8 (26.7)	17 (56.7)	4 (13.3)	1 (3.3)	
Intestinal tract	11	3 (27.3)	5 (45.5)	2 (18.2)	1 (9.1)	
Peritoneum	9	1 (11.1)	2 (22.2)	2 (22.2)	4 (44.4)	
Urinary tract	3	2 (66.7)	1 (33.3)	0 (0.0)	0 (0.0)	

**Table III.** Comparison of biochemical characteristics between the infection and non-infection groups.

Biochemical characteristics	Infection (n=53) n (%)	Non-infection (n=84) n (%)	P-value
Hyperammonemia	49 (92.45)	64 (76.19)	0.015
Increased NEUT	39 (73.58)	8 (9.52)	<0.0001
Hyperbilirubinemia	24 (45.28)	35 (41.67)	0.677
Hyponatremia	40 (75.47)	39 (46.43)	0.001
Low albumin	42 (79.25)	53 (63.1)	0.046
Long PT (%)	34 (64.15)	50 (59.52)	0.588

NEUT, neutrophil; PT, prothrombin time.

**Table IV.** Comparison of etiology at different infection sites.

Infection site	n	Etiology (n, %)
Respiratory tract	30	Pneumococci (12, 40.00)
		*Pseudomonas aeruginosa* (16, 53.33)
		Other (2, 6.67)
Intestinal tract	11	*E. coli* (7, 63.64)
		*Clostridium perfringens* (3, 27.27)
		Other (1, 9.09)
Peritoneum	9	*E. coli* (7, 77.78)
		Other (2, 22.22)
Urinary tract	3	*E. coli* (3, 100.00)
